# Oncoviruses in the Oral Cavity: Recent Advances in Understanding Viral Infections and Tumorigenesis

**DOI:** 10.3390/ijms26146721

**Published:** 2025-07-13

**Authors:** Letícia Bomfim Campos, Ana Carolina Silva Guimarães, Jéssica Gonçalves Pereira, Carla Sousa da Silva, Nathália Alves Araújo de Almeida, Pedro do Nascimento Marinho, Rafaela Moraes Pereira de Sousa, Irena Duś-Ilnicka, Vanessa Salete de Paula

**Affiliations:** 1 Laboratory of Molecular Virology and Parasitology, Oswaldo Cruz Institute (IOC/FIOCRUZ), Rio de Janeiro 21040-900, RJ, Brazil; leticia_bonfim1998@hotmail.com (L.B.C.); anaguimaraes.bio@gmail.com (A.C.S.G.); jessica-gpereira@hotmail.com (J.G.P.); carlasousadasilva27@gmail.com (C.S.d.S.); natybio92@yahoo.com.br (N.A.A.d.A.); pedromarinho.fiocruz@gmail.com (P.d.N.M.); rafaelamoraesfiocruz@gmail.com (R.M.P.d.S.); 2 Regional Hospital of Baixo Amazonas (HRBA), Santarém 68020-000, PA, Brazil; 3 Department of Oral Pathology, Wrocław Medical University, ul. Krakowska 26, 50-425 Wrocław, Poland

**Keywords:** oral cancer, viral tumorigenesis, oral oncoviruses

## Abstract

Oncoviruses, such as Epstein–Barr virus (EBV), human papillomavirus (HPV), and Kaposi sarcoma-associated herpesvirus (KSHV), have been widely discussed for their oncogenic risk. Initially, the oral cavity was disregarded. In recent years, orientation has shifted to the importance of the oral cavity and cancer-related issues via Handbook 19 titled “Oral Cancer Prevention” by the International Agency for Research on Cancer, the WHO Global Oral Health Status Report 2022, and multiple other actions focused on reducing the oversight of this neglected area. Oncoviruses play a significant role in oral cavity malignancies by establishing persistent infections, evading host immune responses, and inducing cellular transformation through the disruption of normal regulatory pathways. Molecular biology and microbiome research have advanced our understanding of the complex interplay between oncoviruses and oral microbiota, demonstrating how coinfections and dysbiosis can enhance viral oncogenic potential. These findings improve the understanding of virus-induced oral cancers and support the development of novel diagnostic and therapeutic strategies. This narrative review focuses on the relationship between oncoviruses and the oral cavity by focusing on how a specific virus triggers tumorigenesis for each of the described viruses and how it affects oral cavity cancer development. Finally, we describe recent advances and future perspectives including vaccines and/or treatment.

## 1. Introduction

**Figure 1:**
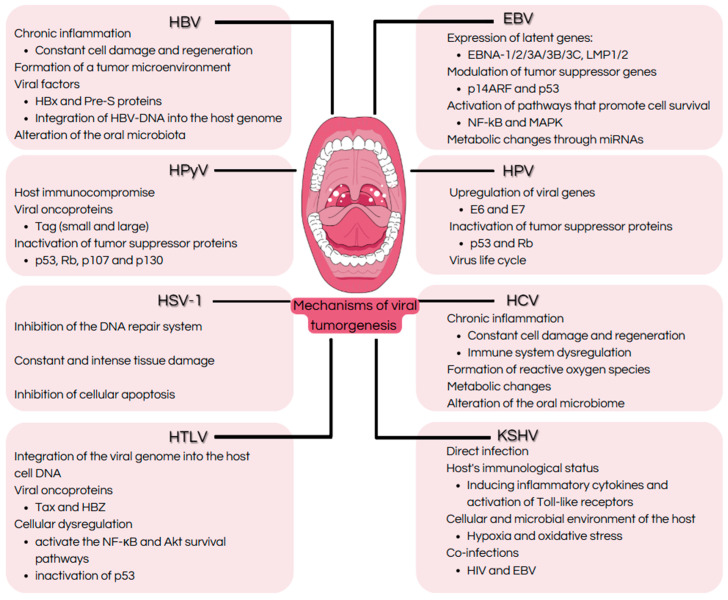
Mechanisms of viral tumorigenesis: Diagram illustrating the different mechanisms by which oncogenic viruses contribute to cancer development. The processes include chronic inflammation, inactivation of tumor suppressor proteins, integration of the viral genome into the host DNA, oxidative stress, metabolic changes, and immunosuppression.

The oral cavity is the second-largest microbial community in the human body and is a complex ecosystem in which diverse microbial agents coexist, including bacteria, fungi, and viral agents, some of which may influence the development of malignancy [1]. Approximately 12% of human cancers worldwide are caused by an infection with an oncovirus; however, the understanding and management of virus-induced cancers still face challenges, especially in the context of the oral cavity [2]. Oncoviruses have been implicated in the pathogenesis of oral cancers including oropharyngeal carcinoma and Kaposi sarcoma. These viruses use intricate mechanisms to evade immune surveillance, establish latency, and induce oncogenesis by modulating cellular pathways that govern cell cycle regulation, apoptosis, and immune responses (Figure 1). Recent advances in virology, microbiome research, and molecular biology have expanded our understanding of the contribution of viral and microbial interactions within the oral cavity to tumorigenesis [1]. Emerging evidence suggests that coinfections and dysbiosis may exacerbate the viral oncogenic potential by activating inflammatory signaling pathways, altering chromatin states, and promoting viral reactivation. Moreover, identifying distinct viral signatures associated with oral cancer provides opportunities for early detection, targeted therapies, and preventive strategies [2].

This review focuses on the viruses classified as carcinogens by the International Agency for Research on Cancer (IARC) due to their established role in human cancers, including those of the oral cavity. Herpes Simplex Virus type 1 (HSV-1) is also included given its prevalence in the oral mucosa and potential co-factor role in oral carcinogenesis, despite not being officially classified as oncogenic.

We explore the latest findings on the role of oncoviruses in the oral cavity, highlighting the molecular mechanisms of pathogenesis, their interactions with the oral microbiota, and their implications for cancer development. We also discuss future perspectives, including the potential for novel diagnostic tools and therapeutic approaches, to mitigate the burden of virus-associated oral malignancies.

## 2. Epstein–Barr Virus

The Epstein–Barr virus (EBV) was discovered in 1964 by Michael Anthony Epstein and Yvonne Barr in African Burkitt lymphoma cells [3]. Therefore, its oncogenic potential was suspected. EBV infection is now known to be endemic worldwide, affecting over 90% of the global population [4]. As a member of the Orthoherpesviridae family, EBV has a large linear double-stranded deoxyribonucleic acid (DNA) genome of approximately 172 kb [5] and two subtypes (EBV-1 and EBV-2), which differ in the loci of the EBV nuclear antigen (EBNA) [6,7]. The virus exhibits two modes of gene expression: a lytic cycle, which involves viral replication and a latent cycle, in which it persists in the host without producing new viral particles.

EBV primarily infects epithelial cells and B lymphocytes and is transmitted through saliva [7]. Primary infection is usually asymptomatic in early childhood. However, if delayed, it can lead to infectious mononucleosis (IM) marked by fever, pharyngitis, and lymphadenopathy [8]. EBV infection often remains latent inside memory B cells, which supports its role in the development of various cancers [9].

EBV is an oncogenic virus classified as a Group I carcinogen by the IARC and is linked to several malignancies. Viral latency has been observed in nasopharyngeal carcinoma (NPC), gastric carcinoma (GC), and oral squamous cell carcinomas (OSCCs). In contrast, lymphoid tissue latency is associated with Burkitt’s lymphoma (BL), Hodgkin’s lymphoma (HL), diffuse large B-cell lymphoma (DLBCL), NK/T-cell lymphomas, and post-transplant lymphoproliferative disease. Additionally, rare cancers such as leiomyosarcoma and primary effusion lymphoma have also been linked to the virus. EBV has been implicated in the development of approximately 1.5% of all cancers worldwide [10].

### 2.1. How EBV Triggers Tumorigenesis

EBV induces tumorigenesis by expressing oncogenic latent genes, which activate signaling pathways such as MAPK, JAK/STAT, NF-κB, TGF-β, Wnt/β-catenina, and PI3K/AKT [11]. Individuals with IM have a 4-fold increased risk of developing Hodgkin’s lymphoma, and high viral loads are correlated with tumor development [12]. Chromosomal abnormalities, mutations, and epigenetic alterations are standard features of oral cancer [13]. EBV initially infects the oropharyngeal epithelial cells and B lymphocytes, transforming them into lymphoblastoid cell lines [14]. In latency stage III, EBV expresses all latent genes, including EBNA-1/2/3A/3B/3C, LMP-1/2, small ribonucleic acid (RNA) (EBER1/2), and microRNAs, and differentiates B cells into proliferating blasts [15]. Then, in latency stage II, after B cell blasts migrate to the germinal center, only LMP1, LMP2, and EBNA1 are expressed [16]. At the same time, the final stage, or latency 0, involves no viral gene expression and differentiation into memory B cells that circulate in peripheral blood [16]. Moreover, latently-infected memory B cells, when stimulated, re-enter the germinal center to differentiate into plasma cells, and the virus is reactivated, returning to its lytic cycle. This leads to viral elimination in the saliva, and the cycle begins again, infecting new naive B cells [17].

Therefore, the expression of EBNA1 ensures viral persistence by binding to the EBV genome and aiding replication [18]. This protein influences gene expression by altering cell cycle regulation and activating oncogenes, such as MYC and NOX2 [19]. EBNA2 is crucial for B cell immortalization. This protein regulates viral and host gene expression by interacting with host transcription factors and EBNALP (a transcriptional co-activator of EBNA2) and regulates chromatin looping and accessibility, gene expression, and MYC induction [20]. EBNA3A/C modulates tumor suppressors, such as p14ARF and p53, promoting cell cycle progression and inhibiting apoptosis [21,22].

The LMP1 protein mimics CD40 receptor signaling, activating NF-κB and MAPK pathways, promoting survival, and inducing immune modulation [11]. LMP1 oncoprotein can activate different classes of MAPKs, such as ERK, JNK, and p38, to reduce the expression of dual-specific phosphatase 6 and 8 (DUSP6 and DUSP8), thereby promoting cell proliferation and increasing the expression of the c-Jun transcription factor, which facilitates the occurrence of epithelial–mesenchymal transition (EMT) [23]. LMP1 can activate JAKs and then activate the STAT dimer, which promotes the corresponding transcriptional regulation in the nucleus and can provide feedback and enhance the regulation of its expression [23]. LMP1 increases the transcription of AP-1 transcription factors through JAK/STAT and promotes the expression of programmed death ligand (PD-L1) to evade immune surveillance [24]. NF-κB dysregulation, often driven by the oncoprotein LMP1, plays a central role in tumor development by promoting proliferation, apoptosis resistance, and metastasis [25]. LMP1 suppresses miR-203, upregulating CDH6 and enhancing EMT [26]. Additionally, it activates the IKK2/TPL2/JNK pathway and induces cell immortalization through hTERT activation and PINX1 inhibition [27].

LMP2A/B mimics host B cell receptor (BCR) signaling, blocks tyrosine kinase signaling following antigen activation of BCR, inhibits viral reactivation, induces DNA methyltransferase activity, enhances cell migration, and inhibits epithelial differentiation [11]. Through the STAT3 pathway, viral proteins LMP1 and LMP2A can activate the homeobox gene HLX to inhibit apoptosis [28]. LMP1 promotes angiogenesis through the PI3K/AKT/HIF-1α/CCL5 signaling axis, and LMP2A promotes it through the PI3K/AKT/mTOR/HIF-1α signaling axis [29,30]. LMP1 inhibits PTEN through miRNA-21 and potentiates the PI3K/AKT signaling pathway to promote cancer stem cell formation and proliferation [31]. LMP2A also inhibits the expression of GSK-3β through the PI3K/AKT signaling pathway, which interferes with the formation of protein complexes and promotes the accumulation of β-catenin in the nucleus to induce EMT [32].

EBV lytic proteins such as BZLF1 and BRLF1 contribute to tumorigenesis by promoting inflammation and angiogenesis via cytokine secretion [33]. Infected cells can promote the activation of the TGF-β signaling pathway and increase the expression of the BZLF1 gene, leading to increased viral replication and enhanced infectivity of EBV [34]. Small RNAs such as EBER assist in immune evasion and are linked to resistance to apoptosis [11]. Additionally, viral miRNAs, including BHRF1, facilitate B cell transformation by targeting tumor suppressor genes such as PTEN and p27 [35]. EBV-miRNA-BART13 inhibits NKIRAS2, thereby promoting cell proliferation, migration, and invasion [36]. EBV-miRNA-BART7-3P can promote the high expression of β-catenin by inhibiting PTEN, leading to EMT [37]. EBV-miRNA-BART13-3P and EBV-miRNA-BART22 inhibit the upstream and downstream MAPK pathways, promote EMT, and resist apoptosis, respectively [38,39].

### 2.2. EBV and Oral Cavity Cancer

Various oral malignancies, both epithelial and lymphoid, are associated with EBV because of its latency in the oral cavity [11]. EBV has a predominant tropism by B lymphocytes and epithelial cells [40]. Endothelial cells of periodontal tissues are commonly infected with EBV, and this infection contributes to the pathogenesis of periodontitis [41]. The virus can replicate in the gingiva, tongue, and tonsillar epithelium [41]. Several oral malignancies are associated with EBV due to its latency in B cells circulating in the blood and lymphoepithelial tissue of the oropharynx, such as tonsils and adenoids, which frequently allows lytic reinfections in epithelial cells of the oral cavity [11].

A two-fold higher chance of EBV detection has been reported in patients with malignant orofacial tumors than in those with benign orofacial tumors [42]. Among epithelial malignancies, the most common oral cancer is OSCC, which can arise in the mucosa of the oral cavity or oropharynx. Several carcinogenic factors have been implicated, such as smoking, alcohol consumption, and human papillomavirus (HPV) infection and co-infection [11,43,44]. Although EBV is detected at elevated levels in OSCC tissues compared to healthy tissues, this association is not yet fully understood as the oncogenic process involves multiple cofactors and alternative genetic pathways [11]. NPC is highly prevalent in southern Chinese and southeast Asian populations and is always associated with EBV; the virus is present in all malignant epithelial cells [45]. No predominance of one of the EBV subtypes was found in patients with NPC; thus, both subtypes are capable of initiating tumor development [46]. Lymphoepitheliomas are undifferentiated carcinomas associated with latent EBV infection, similar to undifferentiated NPC [47], which presents with dense lymphocytic infiltrates. Salivary gland lymphoepitheliomas involving the parotid gland are rare, but a high incidence has been observed among the native populations of Greenland, Alaska, and some Asian populations [48]. All tumors were associated with EBV infection in endemic areas, whereas tumors from non-endemic areas were sporadically associated [49].

EBV-associated oral lymphomas are characterized by the malignant growth of lymphoid cells or precursors [50] that arise in the oral cavity or Waldeyer’s ring, which surrounds the oropharynx and nasopharynx. EBV-related oral lymphomas account for 3% of lymphomas overall and 4% of lymphomas in patients with acquired immunodeficiency syndrome (AIDS) [51]; they include HL, BL, and DLBCL. The latter two lymphomas may present with swelling or ulcerations in areas such as the gingiva, tonsils, tongue, and palate and are often misdiagnosed as periapical abscesses. The genesis of these lymphomas is linked to the interaction between viral gene expression and genetic alterations in the host [11]. EBV is implicated in 20–50% cases of classical HL, which primarily affects the lymph nodes but can also occur extranodally in the oral cavity [52]. BL is a highly aggressive cancer that can be classified as endemic, sporadic, or immunodeficiency associated [53]. The endemic type is common in regions also endemic for malaria [54]. It is a pediatric cancer that presents as an extranodal tumor in various anatomic sites, including the mandible, and is in most cases positive for EBV [55]. The sporadic type, which is rarer, occurs globally and is associated with a lower frequency of EBV [56]. The immunodeficiency-associated variant occurs mainly in patients with human immunodeficiency virus (HIV), with 30–40% of cases being positive for EBV [57]. DLBCL is the most common lymphoma, accounting for 30% of cases, and frequently manifests in extranodal sites, such as the oral cavity, affecting the gastrointestinal tract and other organs [58].

### 2.3. Recent Advances and Future Perspectives in EBV (Vaccines and/or Treatment)

The oral microbiome comprises a diverse array of bacteria, fungi, and viruses. However, the microbiome of patients with oral cancer contains more potential pathogens than that of healthy individuals [59]. The dysbiosis of this ecosystem is closely associated with the presence of certain species of microorganisms involved in oral carcinogenesis, whose interaction favors the occurrence of chronic inflammation, the synthesis of carcinogens, and the disruption of the epithelial barrier integrity [60,61].

*Porphyromonas gingivalis* and *Fusobacterium nucleatum* are important periodontal pathogens in oral squamous cell carcinoma (OSCC), known for their ability to persist intracellularly in epithelial cells, induce chronic inflammation, and promote cellular invasion [62,63]. *P. gingivalis* also disrupts apoptosis through multiple pathways [64]. Increased detection of *Bacteroidetes* in OSCC patients, compared to leukoplakia and healthy controls, suggests its role in tumorigenesis and diagnostic potential [65]. Other bacterial species whose detection in patients with oral cancer has been associated are *Alloprevotella* sp., *Prevotella* sp., *Capnocytophaga* sp., and *Streptococcus* sp. [66]. Under opportunistic conditions, *Candida albicans* induce acute or chronic oral candidiasis [67] and can contribute to OSCC by exacerbating other oral infections and producing carcinogens such as nitrosamines [68,69]. The synergy between bacterial, fungal, and EBV present in the oral cavity occurs due to the promotion of an inflammatory environment and the release of metabolites that cause the reactivation of the latent virus [70,71]. The expression of EBV viral proteins causes the suppression of the immune response and cell proliferation, favoring chronic inflammation, the occurrence of mutations, and the development of cancer [72].

Patients with NPC present with a distinct microbiota with low species diversity. The presence of *Streptococcus sanguinis* was recently associated with increased serum levels of EBV VCA-IgA, the induction of EBV lytic activation via hydrogen peroxide metabolite, the activation of the TNF-α and NF-κB signaling pathways, and the demethylation of the virus genome [73]. *Peptostreptococcus stomatis* ASV.1f05 is associated with carcinoma progression and is overrepresented in the oral microbiota of patients with OSCC [74]. *Capnocytophaga sputigena* ASV.ce54 in the oral cavity of patients with OSCC is associated with tumor recurrence [75]. The prevalence of 56.2% co-infection with EBV, HPV, and polyoma BK virus (BKPyV) was detected in patients with laryngeal, oropharyngeal, and oral cavity cancer [76]. Furthermore, co-infections were more frequent in tumors with larger dimensions and greater lymph node involvement.

Oral cancer treatment typically involves surgery, radiotherapy, immunotherapy, and chemotherapy, either alone or in combination, with surgery aimed at maximal tumor removal; however, this often increases the risk of recurrence and reduces survival rates [77]. Furthermore, radio- and chemotherapeutic treatments are associated with complications in the oral cavity and pharynx, including side effects such as dry mouth, mucositis, oral candidiasis, osteoradionecrosis of the jaw, loss of taste, caries, and periodontal diseases [78]. Preventive measures for oral cancer must focus on increasing public awareness of risk factors, disease severity, and the importance of oral self-examination practices [79].

Other diagnostic and therapeutic strategies for EBV-associated oral cavity cancers include viral biomarkers, antivirals, lytic cycle induction, nano-inhibitors, immune stimulants, and vaccines. Several strategies have been developed for the early diagnosis of NPC using EBV-related biomarkers, including serum antibodies and plasma nucleic acid markers, which have been tested for screening [80,81]. Currently, the detection of methylated EBV DNA may be a promising tool; self-sampling of saliva and/or oropharyngeal swabs showed better diagnostic performance than oral swabs and mouthwashes [82]. A vaccine targeting the EBV 350 glycoprotein (gp350) showed partial success [83]. In a phase 2 trial, the monomeric EBV gp350 vaccine has been shown to reduce the incidence of infectious mononucleosis but not the rate of EBV infection [84]. It has been suggested that the inclusion of gB, gH, and gL in vaccine formulations could be a more effective prophylactic measure against EBV infection [85]. However, no licensed prophylactic or therapeutic vaccines against EBV are currently available, highlighting the need for further research and development.

Alternative therapies such as nano- and phytotherapies are gaining attention owing to their potential in oral cancer treatment [17]. Small-molecule inhibitors that target EBNA1, such as VK-2019, are promising and are undergoing clinical trials for the treatment of advanced nasopharyngeal carcinoma [86]. Additionally, drugs exhibit anti-EBV activity by inhibiting EBV replication, underscoring the need for further research and clinical trials to validate their safety and efficacy [87,88]. Cell therapy involving engineered T-cell receptors and CAR-T cell therapy for EBV proteins is under investigation [89,90]; however, no vaccine or treatment can eliminate latent EBV. Identifying the specific EBV signaling pathways involved in tumor induction and progression is crucial, as is the continued exploration of vaccines and treatments for EBV-associated cancers.

## 3. Kaposi-Sarcoma-Associated Herpesvirus (KSHV)

Kaposi-sarcoma-associated herpesvirus (KSHV), also called human rhadinovirus gamma-8 (HHV-8) according to the most recent report from the International Committee of Taxonomy of Viruses (ICTV), was first described in 1994 by Chang et al. in Kaposi sarcoma samples from patients with AIDS [91]. It belongs to the Gammaherpesvirinae subfamily along with EBV and is the causative agent of several malignancies that occur mainly in immunosuppressed individuals, including Kaposi sarcoma (KS), primary effusion lymphoma (PEL), multicentric Castleman disease (MCD), and inflammatory cytokine syndrome (KICS) [92,93]. These conditions are prone to occur both separately and simultaneously, resulting in essential dysfunction or even death, if not properly diagnosed and treated [93].

HHV-8 is a spherical particle (120–150 nm) consisting of a tightly condensed DNA core, an icosahedral capsid, a tegument, and a glycoprotein-containing lipid envelope [5,94]. Its genome comprises a class 2 linear 170 kb double-stranded DNA (dsDNA) with an approximately 140 kb long unique central coding region (U) flanked by variable numbers of highly GC-rich terminal repeats (TRs) on each side [5,95]. The U-region encompasses a conserved group of genes that primarily encode proteins essential for viral replication, structural components of the virion, and latency. In addition, HHV-8 features a series of genes that contribute to virus-associated diseases, including an array of oncogenic genes that are expressed in both the latent and lytic phases, viral microRNAs, and multiple long non-coding RNAs [96].

The seroprevalence of HHV-8 is significantly lower than that of other herpes viruses and varies from one geographic area to another. The endemic areas are sub-Saharan Africa and the Middle East, where the seroprevalence is between 14% and 86% [92]. In southern Europe, the infection rates vary from 10 to 30%, whereas in the United States, northern Europe, and Latin America, the estimated seroprevalence ranges from 6 to 20% [92,97]. The highest rates were observed in HIV-positive men, with rates reaching 60% in the United States, Europe, and Latin America [92,98,99]. Although there is no consensus on alternative transmission routes, it is well established that saliva is the main transmission fluid alongside semen [92], and behaviors involving exposure to saliva have shown a positive correlation with HHV-8 infection [100]. In addition, there are reports of transmission via blood transfusions in endemic countries, as well as by organ transplantations due to immunosuppressive protocols, leading to aggressive KS development in recipients [92,101].

### 3.1. How KSHV Triggers Tumorigenesis

HHV-8 has a particular tropism for oral and oropharyngeal cells, infecting endothelial and epithelial cells, keratinocytes, monocytes, and macrophages and establishing latency in CD19-positive B lymphocytes, which are abundant in the oral cavity [92,99]. HHV-8 infection has two distinct phases determined by its life cycle. The latent phase, established as the most common infection pathway, is characterized by a very low to no replication rate, with great regulation of viral gene expression, translation of the minimum genes required to maintain long-term infection, and evasion of the immune response through the synthesis of viral proteins that are similar to host antiviral proteins, thus inhibiting antiviral mechanisms [93,102]. After entering the target cell, the latent cycle may be temporary or may last for the remainder of the host’s life. However, factors that trigger the lytic phase are not fully understood [93,103].

Regarding the shift to the lytic phase, current evidence indicates that various cofactors can induce viral activity, replication, and shedding, thereby increasing the risk of HHV-8-associated diseases and facilitating transmission [103]. The most critical factor is the host’s immunological status, followed by viral coinfections (notably HIV and EBV), hypoxia, oxidative stress, and the cellular and microbial environment of the host [92,93]. The interactions between these factors establish a sophisticated gene regulation network involving latency-associated nuclear antigen (LANA) and replication and transcription activator (RTA), the principal promoters of latent and lytic states, respectively [104]. Notably, HHV-8 latency is heavily dependent upon the activation of specific cellular signaling pathways, particularly the NF-κB pathway, which plays a central role in maintaining the expression of latent genes such as LANA, as well as forming a complex with RTA cofactor RBP-Jκ, inhibiting lytic activation by competition. In contrast, HHV-8 reactivation is primarily mediated by mitogen-activated protein kinase (MAPK) signaling cascades [104,105]. These pathways are frequently activated in response to reactive oxygen species (ROS), which serve as indicators of cellular stress and promote the induction of RTA expression [104].

HHV-8 reprograms host metabolism in multiple ways to sustain its replication and promote oncogenesis [96]. It enhances glycolysis by increasing the expression of glucose transporters and glycolytic enzymes, thus promoting energy production and biosynthetic activity in infected cells [106]. During the lytic cycle, HHV-8 downregulates mitochondrial function via viral microRNAs and induces mitophagy through vIRF-1 to prevent apoptosis and ensure viral replication [106]. The virus also manipulates nucleotide biosynthesis by expressing homologs of key metabolic enzymes, ensuring a continuous supply of building blocks for viral genome replication [106]. Additionally, HHV-8 appears to protect infected cells from oxidative stress, contributing to their survival under adverse conditions [106]. In the context of HIV co-infection, host metabolic alterations further support the expansion of HHV-8-infected cells, contributing to more aggressive manifestation [107].

Furthermore, because only a small fraction of individuals who are positive for HHV-8 eventually develop a related condition, with the majority of latent or lytic infections remaining asymptomatic, it is clear that some crucial, yet not fully comprehended, interplay between HHV-8 and these cofactors is necessary to trigger clinical manifestations such as oncogenesis [97,107,108].

KS is the most prominent and well-studied disease associated with HHV-8. KS is a multifocal malignancy that mainly affects endothelial cells and is characterized by the proliferation of vascular spindle cells with substantial angiogenesis and inflammatory infiltration [107]. It primarily affects the skin but can also affect mucosal surfaces or internal organs, ranging from indolent to aggressive presentations [96,109]. The typical clinical presentation is characterized by progressive, multicentric skin lesions that are violaceous, reddish, or brownish, and may manifest as macules, patches, plaques, nodules, or even ulcers. Mucosal involvement may occur in later stages of progression. Nodular lesions may bleed, become hyperkeratotic, and exhibit polychromatic color changes, collarette signs, and white lines, among other dermoscopic findings suggestive of KS [109,110]. It causes significant morbidity and mortality worldwide, with 35,813 new cases and 16,169 deaths reported in 2022 [111]. In 2022, the highest KS incidence rates were reported in countries in Africa (South Africa and Uganda), Latin America (Brazil, Mexico, and Colombia), and Europe (Italy, Russia, and Spain) [111]. KS is broadly categorized into five main epidemiological types. The first is the classic or sporadic form that predominantly affects elderly men of Mediterranean or Eastern European descent. This form typically progresses slowly and is confined to the lower limbs. The second is the endemic African form, which is commonly associated with lymph node involvement in children. The third form, known as the epidemic or AIDS-associated form, became prominent during the AIDS epidemic because of its highly aggressive nature. The fourth type is the iatrogenic form, which arises after organ transplantation due to immunosuppressive treatments designed to prevent organ rejection. Finally, although there is no consensus in the literature regarding this presentation, the fifth is non-epidemic KS, which is associated with HIV-negative, young, and immunocompetent men. The lesions are confined to the skin, localized, and typically show minimal lymphedema [92,97,109,112].

### 3.2. KSHV and Oral Cavity Cancer

Most epidemiological studies on KS or HHV-8 infection have a focus on seroprevalence, whereas data on oral prevalence in the general population remain scarce. This is mainly because such research is often conducted on isolated cases with small sample sizes despite the significant association between the virus and the oral cavity [113]. Nevertheless, these longitudinal and cross-sectional studies have yielded valuable insights into HHV-8 detection in the oral mucosa and its role in the development of KS. The primary site of viral replication is the oropharyngeal mucosa; however, it may also occur within salivary epithelial cells, particularly in immunocompromised patients. Notably, HHV-8 DNA has been reported in 15–57% of seropositive individuals, even during asymptomatic infections [100,114,115].

Oral KS remains the most prevalent AIDS-associated disease, recognized by the World Health Organization (WHO) as one of the seven “cardinal” oral lesions linked to HIV infection in the modern era and is observed in up to 20% of current AIDS patients [99,116]. It develops mainly on the palate, the attached gingiva, and the dorsum of the tongue. Epidemiological studies have shown that 60% of individuals with AIDS-KS will experience, at some point, an oral involvement [99,103]. HIV coinfection synergizes with HHV-8 oncogenesis by depleting the immune system and inducing inflammatory cytokines, Toll-like receptor (TLRs) activation, and synthesizing the Tat protein that promotes HHV-8 replication [103]. Patients with oral KS usually have a lower CD4+ T cell count, indicating advanced immunodeficiency. However, there is no clear correlation between viral oral shedding, tumor development, and CD4+ T cell count, suggesting the impact of other oral microenvironmental factors on oncogenesis [107,117].

Some studies on oral epithelial cultures have shown that epithelial differentiation induces HHV-8 shedding, suggesting the underlying mechanisms for viral shedding on the superficial layers of the oral epithelium. Hence, oral cavity samples usually have the highest viral loads and detection rates among the various sample types [98,117,118].

Furthermore, HHV-8 can infect oral fibroblasts, which may enhance inflammatory cytokines, promoting eventual oral KS development, and abundant tonsillar B lymphocytes to consolidate latent infection [99]. Therefore, oral KS lesions contain higher KSHV loads than skin KS lesions and could signify a worse prognosis for people living with HIV [108,119]. Recent studies have provided evidence for the impact of the oral microbiome on HHV-8-related oncogenesis [107,108].

*Staphylococcus aureus* lipoteichoic acid (LTA) enhances HHV-8 entry and latency oncogene expression by upregulating heparan sulfate proteoglycans, promoting reactive oxygen species (ROS) production, and activating NF-κB and MAPK pathways essentials for HHV-8 latency establishment, as well as posterior replication [104]. Coinfection with *S. aureus* may also facilitate lytic reactivation and suppress Kaposi’s sarcoma tumorigenesis through cyclin D1. Similarly, *Pseudomonas aeruginosa* and *Porphyromonas gingivalis* lipopolysaccharide (LPS) triggers the p38 and JNK pathways, which are involved in lytic reactivation. Additionally, oral KS is associated with reduced microbial diversity, a distinct microbiota signature, and enrichment of Firmicutes, particularly Clostridia, which may drive HHV-8 reactivation and carcinogenesis via histone modification [107,108,120,121,122].

Additionally, *Neisseria gonorrhoeae* infection, an important sexual transmitted agent, has been shown to upregulate the expression of RTA and enhance RTA-mediated gene transcription, amplifying glycolysis and nucleotide synthesis that support HHV-8 lytic reactivation [123]. In the oral cavity, the composition of *Neisseria* sp. communities is highly dynamic and vary by genus, species, and strain. Notably, within localized infection sites such as periodontal pockets, the concentration of bacteria can be substantially higher than their levels detected in broader oral samples [123].

Oral KS is diagnosed through biopsy and histopathological analysis [103]. The current approach to treating AIDS-KS, the most prevalent association, relies primarily on combined antiretroviral therapy (cART) [97]. Mechanistically, cART suppresses HIV replication, reducing chronic immune activation and promoting CD4+ T cell recovery, which in turn helps reestablish immune surveillance against KSHV. Common regimens include integrase strand transfer inhibitors (e.g., dolutegravir and carbotegravir), combined with nucleoside reverse transcriptase inhibitors such as tenofovir and emtricitabine or abacavir and lamivudine. This therapeutic combination not only reduces HIV viral load but also indirectly limits HHV-8 driven oncogenesis by improving host immunity. In advanced or progressive KS, antiretroviral therapy is often complemented by liposomal anthracyclines like doxorubicin or immunotherapeutic agents such as pembrolizumab or pomalidomide to directly target tumor progression [124,125]. However, there is no standardized drug regimen as treatment decisions depend on the tumor microenvironment and other case-specific factors [97,125,126]. Although no universal management strategy exists for KS, its incidence has declined approximately seven-fold since the introduction of cART in 1996 [127].

However, in the early stages of c-ART protocol, KS may progress and even lead to death, possibly triggered by immune reconstitution inflammatory syndrome (IRIS), called Kaposi sarcoma flare [107,109,127]. Furthermore, KS treatment depends on the clinical form and extent of the lesions [92]; for localized lesions with controlled size, surgical excision, intralesional chemotherapy, or sclerosing agents are the preferred treatments, offering fewer side effects, greater convenience, and improving patients’ quality of life. Conversely, for people living with HIV and oral KS, a combination of c-ART is typically the preferred treatment option owing to the aggressiveness of the disease [103]. Oral KS that develops as a part of IRIS should be treated with limited doses of systemic cytotoxic chemotherapy while continuing c-ART [103].

### 3.3. Recent Advances and Future Perspectives in KSHV (Vaccines and/or Treatment)

Some studies have highlighted the importance of histamine in HHV-8 reactivation; thus, future treatments may target histamine receptors or signaling pathways, thereby preventing the lytic phase [92]. Nucleoside inhibitors of viral DNA polymerases, such as ganciclovir, cidofovir, brivudine, or zidovudine, are used to reduce viral shedding and inhibit viral replication, although these drugs have limited efficacy in treating advanced cases [128]. In addition, multiple preclinical studies have focused on identifying other molecular targets to develop therapeutic drugs for HHV-8 infection and associated diseases as well as promising new epigenetic-directed therapeutics that have attracted attention in the cancer research field. Notably, some research studies have shown that polyamine synthesis inhibitors, such as difluoromethylornithine (DMFO), may have a potential usage by addressing LANA expression maintenance, thus inhibiting HHV-8-associated cancer progression [129]. However, the in vivo applications of these drugs in oral infections are yet to be explored. As a result, many future therapeutic targets are being studied, and promising epigenetic drugs are being developed to further decrease the oral KS burden in people living with HIV [99,128].

## 4. Hepatitis B Virus (HBV)

Hepatitis B virus (HBV) was first described in 1947 by McCallum and is called virus B [130], a name that was later changed by the WHO to hepatitis B. HBV belongs to the Hepadnaviridae family, which is composed of hepatotropic viruses with similar morphology and genomic organization [131]. This family comprises five genera: *Parahepadnavirus*, *Metahepadnavirus*, *Herpetohepadnavirus*, *Avihepadnavirus*, and *Orthohepadnavirus*; the latter comprises viruses that infect mammals, where HBV is found [132].

The genetic material of HBV is approximately 3.2 kb and consists of partially double-stranded circular DNA. It is organized into four overlapping open reading frames (ORFs) that encode surface antigens (HBsAg), core antigens (HBcAg), polymerases, and X proteins (HBxAg) [132]. Although HBV is a DNA virus, it has a high mutation rate due to the pre-genomic RNA reverse transcription step [133]. Thus, HBV is classified into 10 genotypes (A to J) and several sub-genotypes distributed worldwide that can present different natural courses [131].

During HBV infection, the nucleocapsid is released into the cytoplasm after the virus enters hepatocytes, and the viral genome is transported to the cell nucleus. Viral DNA is repaired in the nucleus to form covalently closed circular DNA (cccDNA). cccDNA is extremely stable and can persist indefinitely in hepatocytes, acting as a template for the production of subgenomic and pregenomic RNA (sgRNA and pgRNA). sgRNAs are used to synthesize viral proteins. Simultaneously, pgRNA and polymerase are encapsulated in the nucleocapsid, where they are converted into DNA by reverse transcription, contributing to the formation of new viral particles [131].

The WHO estimates that 254 million people live with HBV worldwide; however, only 13% of these have been diagnosed, and less than 3% receive antiviral treatment. In addition, the estimated number of deaths due to chronic viral hepatitis has increased in recent years, with HBV being the main cause (83%). This finding suggests an increase in the number of cancer cases related to chronic viral hepatitis [111].

HBV can be transmitted from the mother to the child at birth, through contaminated sharps, or unprotected sexual contact [134]. The symptoms of acute hepatitis B depend on the age of infection; infants and children are mostly asymptomatic, and the majority of adults (70%) present with non-specific subclinical symptoms that make early identification difficult, such as fatigue, lack of appetite, nausea, vomiting, abdominal pain, and low-grade fever. Only 30% of adults present with jaundice and dark urine samples. The clinical signs include liver tenderness, hepatomegaly, and splenomegaly [134,135].

### 4.1. How HBV Triggers Tumorigenesis

HBV infections acquired in adulthood develop into chronic hepatitis in less than 5% of the cases, whereas childhood infections become chronic in approximately 95% of the cases [135]. It is estimated that 20% of individuals with chronic HBV infection may progress to cirrhosis, and of these, approximately 5% develop hepatocellular carcinoma (HCC) [135]. HCC is the sixth-most common cancer worldwide, with chronic hepatitis B accounting for 50–60% of cases [136].

HBV-associated HCC may involve direct and indirect mechanisms. Indirectly, mechanisms involve chronic inflammation of the liver and a constant cycle of cell death and regeneration, which can promote mutations in the HBV genome and in the hepatocytes [136]. It is seen that the double mutation in the pre-C region (G1896A) and basal core promoter (T1762/A1764) in the HBV genome was associated as a risk factor for the development of HCC. In addition, point mutations, as well as incisions and deletions in the pre-S regions, are common in patients with HBV-associated HCC and are related to the maintenance of the virus in the host cell, promoting cellular transformation and increasing the risk of HCC [137]. Furthermore, due to chronic inflammation, somatic mutations may occur in cells infected by HBV, which may interfere with tumor suppression pathways, such as mutations in the TP53, Axin1, and RB1 genes, or even favor the expression of oncogenes such as CTNNB1, or interfere with cellular resistance to oxidative stress (KEAP1) [137].

Chronic inflammation also generates ROS, which are related to the severity of the disease and the replication status of HBV, and can damage the DNA of liver cells, favoring the occurrence of cancer directly [136]. Furthermore, an association between viral proteins and the induction of ROS production is observed. For example, the structural protein HBx can interact with the outer mitochondrial membrane or interfere with respiratory chain complexes, thus promoting the destruction of mitochondrial membrane potential and inducing ROS production [137].

HBV DNA integration is an accidental event in only approximately 1% of hepatocytes during the early stages of infection and is not necessary for viral replication. However, 90% of HBV-related HCC cases show DNA integration, indicating a strong advantage of hepatocytes containing integrated HBV DNA in cancer progression [136]. Although the site of this integration of the viral genome occurs randomly; in some cases, it can reach regions of human DNA that favor cell growth, favoring genomic instability, the occurrence of mutations, and the loss of chromosomal fragments. Several genes are recurrent targets of HBV integration, and the most studied are TERT, MLL4, CCNA2, CCNE1, and FN1, which are involved in signaling pathways associated with cancer. The TERT (telomerase reverse transcriptase) is the most common site of HBV integration. When HBV regulatory sequences integrate close to or within TERT, there is an abnormal activation of telomerase. This allows the cell to continue replicating indefinitely, which favors malignant transformation. Among the viral genes most frequently integrated into the host cell DNA are the HBV X and C genes. X gene integration is particularly important as it can directly activate oncogenes such as Myc, Ras, Src, and CyclinD1, as well as inhibit suppressor genes such as P53 and Rb [137].

Metabolic alterations have been shown to be important for tumor development. The liver plays a key role in carbohydrate, protein, and lipid metabolism, and alterations in these pathways have been associated with the development of HCC. During HBV infection, both viral replication and virally encoded proteins, especially HBx and its truncated form (Ct-HBx), contribute significantly to this metabolic reprogramming, affecting not only tumor cells but also shaping the tumor microenvironment. HBx, for example, stimulates glycolysis and fatty acid oxidation, promoting survival and stem cell characteristics in the tumor [138]. However, the mechanisms associated with HCC development during HBV infection are not yet fully elucidated.

### 4.2. HBV and Oral Cavity Cancer

HBV has been identified in saliva and gingival crevicular fluid (GCF) samples, including viral DNA and HBsAg. One hypothesis for the presence of HBV in saliva is that when circulating in the bloodstream, the virus can reach the lymphatic system, the GCF, and, consequently, the saliva. Another hypothesis highlights the occurrence of gingival bleeding (visible or not), allowing the virus to be detected in the saliva [138].

In addition, HBV infection is related to changes in the cytokine profile of the saliva, which can lead to oral inflammatory diseases. The presence of HBV in the oral cavity is associated with a high risk of periodontal disease [138]. Furthermore, HBV infection causes changes in the oral microbiota, thereby increasing the risk of opportunistic oral infections [139]. Patients with HBV-induced chronic liver disease have lower bacterial diversity in the oral microbiota, such as increased Firmicutes and Spirochaetes and reduced Actinobacteria [139]. Oral microbiome dysbiosis may be involved in the progression of liver disease to cirrhosis and liver cancer since opportunistic oral bacteria, as well as their products, can migrate to the intestine and cause changes in the intestinal microbiota, which can, through the portal circulation, reach the liver, influencing liver complications. This relationship is called the “oral–gut–liver” axis [140]. Furthermore, for example, an increase in bacteria from the Firmicutes and Actinobacteria phyla is observed in patients with HCC associated with HBV, which may even be a biomarker for early diagnosis of liver cancer [141].

The relationship between chronic HBV infection and development of liver cancer is well established. However, HBV infection is also associated with the development of extrahepatic tumors, particularly in areas with a high prevalence of the virus [142]. Several studies have shown a correlation between HBV infection and the occurrence or worse outcomes of head and neck cancer [143,144,145,146,147]. Takata et al. (2002) and Song et al. (2019) reported a higher prevalence of HBsAg in patients with oral tumors [148,149]. The HBsAg is the main serological marker of active HBV infection [134].

The biological mechanisms underlying extrahepatic cancers are yet to be fully elucidated. Some studies have suggested that several mechanisms may be involved, such as immune dysregulation caused by chronic HBV inflammation, which promotes the creation of a tumor microenvironment [142]. In addition to direct factors, such as the detection of viral DNA and X protein in extrahepatic tissues, these factors can initiate and promote tumorigenesis in these tissues [142,149,150,151,152].

### 4.3. Recent Advances and Future Perspectives in HBV (Vaccines and/or Treatment)

A safe and effective vaccine against HBV has been available since 1981, and universal vaccination of infants has led to a decrease in the prevalence of HBV and incidence of HCC in various parts of the world. However, vaccination coverage varies globally, with 90% in the Western Pacific and Americas and 56% in Southeast Asia, making it difficult to eliminate hepatitis B [134]. The treatment for HBV infection is systemic, using drugs administered orally; however, there is no specific treatment for HBV infection in the oral cavity. The main goals of current HBV therapies are sustained suppression of viral replication and hepatocyte inflammation as current therapies cannot eliminate cccDNA in the nucleus of infected cells [134]. Several therapies are in clinical trials to treat hepatitis B, including direct-acting antivirals targeting different stages of the HBV cycle, immune modulators, and gene editors aimed at eliminating cccDNA [153,154].

## 5. Hepatitis C Virus (HCV)

Hepatitis C virus (HCV) was first described in 1975 by Feinstone et al., who reported cases of post-transfusion hepatitis that were not associated with hepatitis A virus (HAV) or HBV [155]. HCV belongs to the *Flaviviridae* family, which comprises the genera *Flavivirus*, *Pegivirus*, *Pestivirus*, and *Hepacivirus*, with HCV belonging to the latter [156]. The HCV genome comprises a single-stranded RNA (ssRNA) with a positive polarity of approximately 9.6 kb long. It has a single ORF that codes for the viral polyprotein, which is subsequently cleaved into three structural proteins (core, E1, and E2) that form the viral particle and seven non-structural proteins (p7, NS2, NS3, NS4A, NS4B, NS5A, and NS5B) that participate in the viral replicative cycle [157]. HCV is characterized by high genetic diversity and is currently classified into eight genotypes (named 1–8), which can be further subdivided into numerous sub-genotypes [158].

According to WHO data, an estimated 50 million people worldwide are chronic HCV carriers; however, only 36% of these have been diagnosed, and 20% have received antiviral treatment, showing that the elimination of hepatitis C is still a challenge [111]. HCV transmission occurs mainly via the parenteral route; however, vertical and sexual transmission can also occur, although it is not considered sexually transmitted [157].

Symptoms of acute HCV infection occur in around 15–30% of infected individuals. They are initially non-specific and can include fever, vomiting, fatigue, headache, bone pain, weight loss, epigastric pain, and, in some cases, jaundice [159,160]. The development of chronic hepatitis C is related to the age at which the individual acquires the infection, with only 25% of adults naturally achieving viral clearance. Thus, 75% of individuals develop chronic hepatitis C, which is characterized by the persistence of viral RNA for more than six months. It is estimated that 15% of these individuals may develop cirrhosis, and, of these, 1–3% may progress to HCC [159].

With the introduction of direct-acting antivirals (DAAs), approximately 95% of individuals with chronic hepatitis C achieve a sustained virological response (SVR); however, eliminating the virus reduces but does not eliminate the risk of developing liver cancer, especially in cirrhotic individuals. In addition, approximately 150,000 people died in 2021 because of HCV-dependent HCC [161]. This indicates that it remains a public health problem requiring attention.

### 5.1. How HCV Triggers Tumorigenesis

Unlike other oncoviruses, HCV does not integrate its genetic material into the host genome, such as retroviruses, or form episomes. Therefore, HCV-associated HCC carcinogenesis is related to direct and indirect factors in which chronic hepatocyte inflammation leads to the establishment of a tumor microenvironment that favors HCC development [161]. Constant cell regeneration due to the inflammatory process increases the chances of mutations. The constant destruction of infected hepatocytes, mediated by the immune system, leads to the activation of liver regeneration mechanisms, which favors cell proliferation in a highly pro-oxidant environment. During this process, there is an increase in the production of ROS by the immune cells and hepatocyte mitochondria, resulting in DNA damage, genetic mutations, and chromosomal instability, factors that contribute to the malignant transformation of liver cells [161,162].

HCV viral proteins can interfere with tumor suppressor pathways, such as p53 and Rb, and activate oncogenic pathways, such as PI3K/Akt, RAF/MAPK/ERK, and Wnt/β-catenin. NS5A, for example, inhibits apoptosis by blocking caspase activation and interfering with the action of TGF-β, a factor with antiproliferative functions. Persistent activation of the β-catenin pathway by Core and NS5A, either by inhibition of protein degradation or by direct interaction, leads to the expression of genes such as Myc and Cyclin D1, which stimulate cell proliferation [161,162].

Moreover, HCV influences and promotes metabolic and epigenetic changes that favor the development of liver cancer. The virus induces the expression of methyltransferases (such as DNMT1 and DNMT3B) and alters histone acetylation, affecting the expression of tumor suppressor genes such as CDKN2A, RASSF1A, and SOCS1. These epigenetic effects can be mediated directly by viral proteins, such as the Core protein, or indirectly, by metabolic alterations and secretion of non-coding RNAs. These epigenetic modifications are stable and persist even after the infection is cured, which supports the theory that HCV acts as a “hit-and-run” virus—that is, it initiates the process of malignant transformation but does not need to be present at the time of tumor progression [161,162].

HCV interferes with host lipid metabolism, inducing the development of hepatic steatosis, which, in turn, is associated with the development of HCC. The steatosis and insulin resistance, in turn, promote a chronic inflammatory and antiapoptotic state, which favors cell proliferation and immune evasion. In experimental models, the presence of the NS5A protein was sufficient to induce tumors when combined with factors such as a high-fat diet or alcohol [161,162].

Although HCV eradication with DAAs significantly reduces liver disease progression, the epigenetic, metabolic, and immunological scars left by chronic infection remain as risk factors for the development of HCC. This justifies the recommendation of continued surveillance, especially in patients with advanced fibrosis, even after virological cure.

HCV can induce various extrahepatic manifestations, generally associated with the inflammatory process, due to the host immune response against the viral pathogen. However, the presence of HCV antigens, the viral genome, and replicative sequences has already been identified in various other tissues, such as peripheral blood cells, kidneys, pancreas, skin, salivary glands, and oral mucosa [163]. This can lead to the development of tumors in these tissues [163,164].

### 5.2. HCV and Oral Cavity Cancer

HCV RNA and anti-HCV antibodies have been detected in saliva and GCF samples. The mechanisms underlying the presence of HCV in other bodily fluids are not well understood [165]. One hypothesis is that owing to the systemic inflammatory process generated by the chronicity of the disease, gingival bleeding (visible or not) occurs, where viral RNA can be detected. Another hypothesis is that the virus reaches saliva through the GCF as it is also found in this fluid. Finally, HCV has lymphotropism and can be found in mononuclear blood cells, thus being found in fluids that contain these cells, such as saliva [165]. It is essential to highlight that, although HCV is found in other body fluids, transmission of the virus occurs only through the parenteral route.

Furthermore, chronic HCV infection influences oral health, leading to the worsening of periodontal disease [165]. This relationship appears to be associated with indirect infection factors and not with the presence of the virus in the oral cavity. One hypothesis is that the maintenance of a pro-inflammatory environment due to chronic infection alters the balance of the oral microbiota, leading to an increase in pathogenic bacteria, and, consequently, an inflammatory reaction in the oral cavity, favoring periodontal disease [165].

Another hypothesis is that individuals with chronic HCV infection develop insulin resistance (IR) as liver inflammation leads to metabolic changes. Periodontal disease is a systemic condition related to metabolic syndromes such as IR. Therefore, oral diseases may occur owing to metabolic dysregulation caused by HCV [165].

Although periodontal disease is an indirect consequence of HCV infection, conditions such as oral lichen planus (OLP), Sjögren-like sialadenitis, and OSCC are considered extrahepatic manifestations of HCV infection and directly related to immunological alterations caused by the virus [165,166]. However, the mechanisms underlying chronic HCV infection and the occurrence of these oral manifestations have not yet been fully elucidated.

OLP is a chronic inflammatory disease that affects the oral mucosa, causes lesions, and is associated with the risk of developing OSCC, which, in turn, has been reported to be associated with HCV [166]. Sjögren’s syndrome is an autoimmune disease that affects the exocrine glands, including the salivary glands [166]. Although rare, Sjögren’s syndrome can influence the development of B-cell non-HL, a pathology that is also associated with HCV and may, therefore, be a risk factor for its development [163,167].

Several studies have shown an association between HCV infection and the development of head and neck tumors [146,147,168,169]. Borsetto et al. (2020) conducted a meta-analysis and found a significant association between HCV infection and the development of cancers of the oral cavity, oropharynx, and larynx [170]. Rangel et al. (2018) found a higher frequency of HCV infection in individuals with head and neck squamous cell carcinoma (HNSCC) than in those with other types of head and neck cancer. They also observed no difference in the survival time between infected and uninfected patients [171]. These data highlight the importance of HCV infection in the increased risk of developing head and neck tumors and the need to pay attention to these patients.

### 5.3. Recent Advances and Future Perspectives in HCV (Vaccines and/or Treatment)

Current therapy with DAAs represents a significant step forward in the elimination of HCV since the SVR rate is over 90%, regardless of the viral genotype, and the treatment time, as well as the adverse effects of therapy, is much shorter when compared to therapy with interferon and ribavirin, which facilitates adherence to treatment [172]. However, some challenges are still present, such as the existence of non-responders who require different combinations of treatments and the treatment of patients with decompensated liver disease. The main challenge is the diagnosis of HCV-infected individuals as it is only possible to identify them [172].

Although progress has been made with DAAs, there is still a need for an effective vaccine to eradicate HCV infections, especially in at-risk populations. The antibodies generated by primary HCV infection do not guarantee protection against secondary infection, mainly because of viral genetic variability [173].

## 6. Human Papillomaviruses (HPVs)

Human papillomaviruses (HPVs) belong to the family *Papilomaviridae* and are non-enveloped dsDNA viruses that are approximately 50–60 nm in diameter [174]. The virion contains 72 capsomeres and copies of pentameric monomers composed of five identical L1 proteins that assemble into L2 proteins. The expression of recombinant L1, with or without L2, favors the self-assembly of identical virus particles. The HPV genome contains eight reading frames (E1, E2, E4, E5, E6, and E7) and two late regions (L1 and L2) [175]. The reading frames allow replication and transcription, whereas the late regions package the amplified genome into a virion, forming an icosahedral capsid. Viruses move from the cell through natural desquamation, which infects epithelial cells. HPV causes cancer by upregulating E6 and E7 reading frames, which inactivate p53 and Rb in host cells, leading to cellular transformation [176].

### 6.1. How HPV Triggers Tumorigenesis

The association between HPV and oncogenesis was first elucidated 40 years ago by Hausen, who discovered an interaction between the virus and cervical cancer development [177]. Subsequent studies have confirmed the relationship between several types of HPV and other epithelial tumors. Approximately 200 subtypes of HPVs have been registered, most of which can cause human infections [178]. HPV is classified according to its oncogenic potential as low-risk or high-risk HPV. HPVs have a tropism for the differentiated squamous epithelium, manifesting mainly in skin, tegumentary, and mucous membrane infections. HPV infection is associated with cervical cancer in women. Other anogenital neoplasms, including anal, vaginal, vulvar, and penile cancers, have also been associated. HPV is also largely responsible for tumors that arise in the oropharynx and cutaneous warts [179].

HPV transmission occurs through sexual, skin-to-skin, or skin-to-mucosa contact and is the most common sexually transmitted infection worldwide. It is believed that 80% of sexually active adults are exposed to HPV at some point in their lives [180]. These viruses are rarely vertically transmitted during the perinatal period. The life cycle of the papillomavirus is directly related to the differentiation of the host’s epithelial tissue; therefore, the infection can disappear within two years after contact, just as the persistence of the infection can trigger several diseases, including genital warts and malignant neoplasms in the head and neck [181].

HPV is a high-risk factor for a subset of head and neck squamous cell carcinomas, particularly those of the oropharynx [181]. However, other well-established factors, such as tobacco and alcohol consumption and poor oral hygiene, significantly influence the development of neoplasms in this segment [182].

Studies show that the prevalence of high-risk HPV infections is significantly higher in men compared to women, with men who have sex with men having even higher rates [183]. HPV-positive tumors are more frequently reported in individuals who are younger, Caucasians, and have higher purchasing power. Considering the mode of HPV transmission of human papillomaviruses, patients with HPV-positive head and neck cancers tend to have multiple sexual partners throughout their lives. They were more likely to have oral, genital, or coronal contact. HPV-16 and HPV-18 are the main HPV subtypes of human papillomavirus responsible for most head and neck cancers worldwide [184].

Malignant head and neck neoplasms associated with HPV infections tend to have fewer smaller primary tumors, larger cystic lymph nodes, better performance statuses, and are more likely to have a better prognosis than other forms of head and neck malignancies, thus requiring reduced treatment to minimize side effects [185]. However, a subset of HPV-related neoplasms do not respond well to treatment and require a better understanding of the tumor and more extensive therapeutic interventions [186].

Approximately 40% of the individuals with head and neck cancer initially develop localized early-stage disease [187]. Treatment of these patients can be performed using a single modality of therapy, such as surgery with curative intent or radical radiotherapy, depending on the location of the tumor. For patients with locally advanced tumors, multimodal therapy is most commonly indicated, and if the patient initially undergoes surgery, postoperative radiotherapy with or without chemotherapy should be considered. Concomitant radiotherapy with chemotherapy is also widely considered, although it is not the best initial approach [186].

### 6.2. HPV and Oral Cavity Cancer

HPV, observed to cause genital and anal cancer in the last decade, has been recognized as an etiological factor for a set of tumors in the oral cavity. The relationship between HPV and carcinogenesis in this region reveals that the virus plays an important role in the development of HNSCC. HNSCC is a group of neoplasms that develops anatomically in the oral cavity, oropharynx, hypopharynx, larynx, and nasopharynx [182].

Although risk factors such as tobacco and alcohol consumption and poor oral hygiene are well known and accepted for the occurrence of oral cancer, the role of HPV as a risk factor for HNSCC remains controversial [188].

Oncoviruses are recognized as oral infections that affect sexual behavior and are linked to a significant number of sexual partners throughout life and sexual behavior among men. In addition, sexual practices, specifically how oral sex favors infection of the oropharyngeal mucosa, increase the risk of developing neoplasms [189]. In the last few decades, the number of HPV-related tumors has increased significantly in developed countries. Thus, it is believed that the papillomavirus infection in humans can overcome the risk factors that were previously more influential [190].

HPV is classified according to its oncogenic capacity; therefore, it is classified as either low-level HPV risk or high risk (HR). Approximately 12 HR-HPV genotypes have been identified in oral cavity tumors: HPV16, 18, 26, 31, 33, 35, 45, 56, 58, 59, and 67 [191]. Predominance of HPV16 accounts for 86% of the viruses found in HNSCC [192].

Among the various regions of the head and neck susceptible to infection by human papillomavirus, the most clinically significant site is the oropharynx. Interestingly, HPV shows a particular tropism for the reticulated epithelium of the tonsillar crypts and the base of the tongue, where HPV-induced oropharyngeal squamous cell carcinoma predominantly arises [193,194].

The relationship between HPV and the oral microbiota may be crucial in the pathogenesis of HPV-associated oral tumors as the oral microbial flora—composed of bacteria, fungi, and viruses—contributes to the regulation of both oral and systemic health. A simple imbalance of the microbiota can trigger inflammatory reactions, DNA damage, apoptosis, and altered metabolism, creating conditions for malignant transformation, anti-apoptotic activity, and carcinogen secretion. Furthermore, it can influence HPV viral persistence, immune response, and the tumor microenvironment, impacting disease progression and treatment outcomes. It is important to emphasize that HPV alone is not capable of causing tumors; it necessarily requires associated factors such as immune regulation, microbial environment, mucosal secretions, and tissue integrity [193].

### 6.3. Recent Advances and Future Perspectives in HPV (Vaccines and/or Treatment)

The relationship between HPV and the development of carcinoma in the oral cavity has been recognized relatively recently; however, the characteristics of HPV-positive patients differ from those of patients typically affected by other head and neck tumors. This association between the virus and oral cancer represents a public health problem as more young men who are subject to infections make illnesses transmissible, although the prevalence of cases points to individuals of the same sex over 40 years of age. Vaccination is recognized as one vaccine that prevents HR-HPV subtypes and reduces viral circulation in the oral cavity. Therefore, health professionals must warn the public about the nuances involved in the relationship between HPV and carcinogenesis [195].

## 7. Human Polyomaviruses (HPyVs)

The name polyomavirus is derived from poly-(Greek; multiple) and -oma (Greek; tumors) and was named when the murine polyomavirus was first discovered in immunocompromised neonatal mice with multiple tumors in 1953 [196]. Further studies have shown that many polyomaviruses have the potential to mediate cell transformation and tumorigenesis in various experimental models [197].

Human polyomaviruses are ubiquitous and opportunistic in nature and are rapidly expanding viral clusters [197]. The *Polyomaviridae* family consists of 14 polyomaviruses, three of which infect humans: BKPyV (*Betapolyomavirus secuhominis*), John Cunningham (JCPyV), and MCPyV (*Alphapolyomavirus quintihominis*) [198]. These viruses are small (45 nm), non-enveloped, and have capsomers with icosahedral symmetry [198].

The genome of polyomaviruses consists of a circular dsDNA of approximately 5000 bp [197,198]. The BKPyV and JCPyV genomes have been used in previous studies because of their extremely conserved coding regions [198]. JCPyV and BKPyV share approximately 70% similarity in their genomes, whereas MCPyV has the largest genome (5387 bp) [198]. The genome is divided into three different regions: (1) the early gene region, which includes large T and small T antigens and regulatory proteins responsible for viral transformation, replication, and regulation of gene expression; (2) the late genes, which encode capsid proteins VP1, VP2, and VP3; and (3) the noncoding regulatory region [198].

Polyomaviruses are prevalent worldwide, with primary infections occurring in childhood, but can occur at any age [199]. These viruses establish persistent infections, usually in early life, and remain latent in several body compartments, including the tonsils, lower urinary tract, lymphoid tissues, and bone marrow [199]. Kidney involvement with viremia is frequently observed in immunodeficient patients undergoing renal transplantation [199,200]. The exact route of polyomavirus transmission is poorly understood and remains unclear. JCPyV, BKPyV, and SV40 (simian polyomavirus) are frequently detected in urine and can play a role in the transmission of these viruses [199]. JCPyV and BKPyV were found in tonsillar tissues, consistent with respiratory transmission or transmission via oral and respiratory fluids [199].

Typically, polyomavirus infections are asymptomatic or are associated with mild pathological changes in the respiratory and urinary tracts of immunocompetent individuals [199]. BKPyV is frequently associated with kidney infections and is common in renal transplants, resulting in nephropathy or cystitis [199]. There have been some reports of disseminated BKPyV causing meningitis, retinitis, pneumonia, or vasculopathy [199]. JCPyV can be associated with neurological symptoms; this virus can infect and destroy oligodendrocytes of the central nervous system, causing a fatal demyelinating disease called progressive multifocal leukophalopathy (PML) [199].

### 7.1. How HPyVs Triggers Tumorigenesis

According to the IARC, among the known members, MCPyV is classified as a Group 2A carcinogen (probably carcinogenic), whereas JCPyV and BKPyV are classified as Group 2B carcinogens (possibly carcinogenic to humans), viruses that have been frequently associated in human cancers [201]. The process of tumorigenesis transformation is mediated by these viruses driven by viral pleiotropic regulatory proteins called T antigens [197]. The T antigens target cellular regulatory factors to favor cell proliferation, immune evasion and downregulation of apoptosis, by the association with p53 [197].

Extensive studies on SV40 have identified two major viral oncoproteins, large Tag (Tag) (90–100 kDa nuclear protein) and small tag (tag) (17–22 kDa), present in all HPyV [196,197,199,200,202]. Tag oncoproteins are used in cellular transformations to bypass viral cellular checkpoints [196]. The host cell is forced into the S phase of the cell cycle, bypassing apoptosis by inactivating the tumor suppressor p53 via tag binding [196]. Other small protein members, such as RB, p107, and p130, also bind large tags that prevent interaction with E2F1, which is an important transcription factor responsible for the controlled expression of cell-cycle-promoting genes, causing the loss of suppression of cell replication [196]. In this complex mechanism, the binding of Tag to p53 and pRb leads to highly proliferative and uncontrolled cell growth [196]. The small-tag oncoprotein is an oncogenic enhancer that interacts with the tumor suppressor serine-threonine protein phosphatase SA (PP2A), which mediates transformation-enhancing signaling pathways. Large and small tag oncoproteins contribute to the tumorigenic potential of all human polyomaviruses [196].

The association of MCPyV and cancer was first reported by Chand and Moore by the first isolation of this virus in Merkell cell carcinoma (MCC) [203]. In this study, they found in all tumor samples the viral DNA integrated into the cancer cell genome, demonstrating that the integration was an early event during oncogenesis, occurring before the expansion of the tumor cells [204]. Studies demonstrated that 80% of all MCC contained MCPyV integrated in tumor cells [204].

The MCPyV has the potential to integrate in random sites by accidental genome fragmentation during viral replication, leading to mutational events in the viral genome, prompting carcinogenesis [204]. The viral genome is compromised by 5387 bp and expresses the large T (LT), small (sT), and 57kT antigens [197]. Tumors expressing both LT and sT antigens are considered as major viral oncogenes responsible for driving MCPyV-associated tumor development [204]. However, while the sT antigen was expressed in its normal (wild-type) form, the LT antigen appeared in a truncated version (tLT) lacking its C-terminal portion [197]. Similar to other human polyomaviruses, the tLT of MCPyV retains important regions at its N-terminus, such as the J domain and the LXCXE motif, which enable it to bind and inhibit the retinoblastoma protein (pRb) [197]. In addition, tLT contains an origin-binding domain, a nuclear localization signal (NLS), and a helicase-binding domain but lacks the region responsible for binding p53 [197].

Experimental studies demonstrated that silencing LT in MCC cells halts their growth [205]. This effect cannot be reversed by a mutant version of LT that lacks the ability to bind pRb, reinforcing the essential role of the LT-pRb interaction in the development of these tumors [205].

The potential oncogenicity of JCPyV has not yet been clearly established. Some studies have described an association between JCPyV and tumors in immunosuppressed patients [197]. Moreover, JCPyV DNA has been detected in some gastrointestinal tumors [206]. In tumor cells, JCPyV can cause anchorage-dependent growth, quickly mitosis, prolonged life span, increased ploidy, unstable multicentric chromosomes, centric and acentric rings, dysregulated genomic stability and DNA repair, and increased micronuclei formation [207].

In addition to the viral capsid proteins mentioned, JCPyV expresses three proteins with regulatory functions as large T-Ag and small t-Ag encoded in early region and agnoprotein encoded in the late region [208]. These proteins are involved in interactions with host proteins and dysregulating the cellular processes that lead to changes that may be involved in malignant transformation [208].

Some authors demonstrated that JCPyV T-Ag also interacts with pRb and p53. The association with pRb is thought to activate E2F transcription factors that promote cell cycle progression, whereas the interaction with p53 is to compromise its protective role against both DNA damage and oncogenic transformation [208]. Although, JCPyV T-Ag can modulate signaling proteins in addition to pRb and P53, driving to binds to insulin receptor substrate 1 (IRS-1) and causes its translocation to the nucleus [208]. This process contributes to malignant transformation in medulloblastomas, the most prevalent solid brain tumor in childhood [208].

The oncogenic activity of BKPyV is well demonstrated in vitro and in vivo experimental models. The process of transformation modulated by this virus can be mapped in the early region of the BKPyV genome, which encodes the TAg and tAg [209]. These viral proteins induce alterations in the normal cell cycle, leading to cell immortalization and neoplastic transformation [209]. However, as in the other polyomaviruses, the TAg also can interact with pRb and p53, resulting in the inactivation of this protein and interfering with the response to DNA damage and inducing the unscheduled onset of the S-phase [209].

A characteristic of BKPyV in tumorigenesis is the capacity to TAg to drive the cell to override key cell cycle checkpoint, favoring the accumulation of genetic alterations during each cell replication cycle [209]. The interaction of TAg and pRb leads to the release and nuclear translocation of the E2F factor, inducing quiescent cells to enter the S-phase [209]. The small BKPyV t-Ag plays an important role in transformation by PP2A, an essential tumor suppressor in numerous death-signaling pathways [209].

### 7.2. HPyVs and Oral Cavity Cancer

Oral cancer is the most common malignant neoplasm of the head and neck region and the 16th-most common cancer worldwide [210]. The literature describes the association between polyomaviruses and oral cavity cancers. Polyomavirus infections have been associated with up to 50% of the head, neck, and oral cavity cancers [211].

The viral replication of polyomaviruses demonstrates specific cellular tropisms, although some evidence suggests the interaction of these viruses in oral cavity [196,212,213,214]. The exact mechanism of infection and persistence in oral tissues remain not completely understood; however, some authors have demonstrated the presence of these viruses in epithelial and mesenchymal compartments [214].

The MCPyV showed a primary tropism for dermal fibroblasts and Merkel cells, mainly in mechanoreceptor cells located in the basal layer of stratified epithelia, including gingiva and buccal mucosa [215]. The viral detection of MCPyV in oral squamous cell carcinoma (OSCC) tissue samples demonstrates that this association is more common in keratinized sites of the oral cavity, such as the hard palate and gingiva, presenting a preferential viral association with these tissues [215].

JCPyV, previously classified as neurotropic virus, has also been detected in oral cavity, especially in richly innervated areas such as the dorsal surface of the tongue and the soft palate, demonstrating some tropism with these sites [202]. The JCPyV replicates in epithelial cells of the oral mucosa, more frequently in non-keratinized areas, whose tissue permeability may facilitate viral entry [202]. The same aspect is observed in BKPyV; previously this virus was classified restricted to infect urothelial cells and with potential detection at kidney tissues [216]. Although studies reported its presence in squamous epithelial cells of oral lesions, the viral detection was low, suggesting a subclinical or transient infection; this may be limited to regions such as the minor salivary glands and the inner labial mucosa [202].

In addition to these viruses being detected in the oral cavity, polyomaviruses also indirectly interact with the oral microbiome, modulating immunological and inflammatory microenvironment [217]. Viral infections can influence the oral microbiota by disrupting the homeostasis, leading to dysbiotic conditions that support the proliferation of disease-associated bacterial species [217]. Some authors indicate that the presence of polyomavirus in inflamed mucosae can potentiate the local-immune response, increasing production of pro-inflammatory cytokines such as IL-6 and TNF-α and stimulating pathways that promote cell proliferation and resistance to apoptosis [218]. Persistent inflammation is recognized as a contributor to the initiation and progression of various carcinomas, including OSCC [218].

To date, viral and bacterial coinfection can result in synergistic effects that can induce the effect of cell damage and oxidative stress, a role of conditions that facilitate mutations and oncogene activation [219]. Another critical aspect is the modulation of innate and adaptive immunity by the virus-microbiome interaction. Polyomaviruses have developed mechanisms to prevent host immune defenses, such as disrupting antigen presentation and impairing the function of effector T cells, which aids in long-term persistence [220]. This immune evasion maintains a microenvironment permissive to carcinogenesis [220].

### 7.3. Recent Advances and Future Perspectives in HPyVs (Vaccines and/or Treatment)

No specific antiviral treatments or vaccines are available for polyomaviruses. Treatments are largely experimental or supportive because no universally effective antivirals are available. Antiviral compounds can be targeted to inhibit the replication of polyomaviruses [221]. A major group of drugs has been used in polyomavirus infections as attachment inhibitors, uptake inhibitors, uncoating inhibitors, genome replication inhibitors (LTag, ATPase inhibitors, topoisomerase inhibitors, nucleotide/nucleoside analogs, de novo purine/pyrimidine synthesis inhibitors, protein kinase/phosphatase inhibitors, and nuclear transport inhibitors), and drugs with unknown modes of action [221]. All these classified drugs and drug candidates have limited efficacy during the course of infection [221].

Another treatment strategy involves the reduction of immunosuppression. This treatment presents challenges, particularly for recipients of stem cell transplants (SCTs) with active graft-versus-host disease [221]. Patients with SCT and compromised immune systems experience persistent cytolytic viral replication and unresolved symptomatic BKPyV infection [221]. Treatment using a source from the pooled plasma of numerous donors (IVIG) harbors neutralizing antibodies against BKPyV and is associated with a 90% reduction in infection in vitro [221]. Unfortunately, the efficacy of IVIG therapy is complicated by factors such as widely variable dosing, lack of information on neutralizing antibody titers, and initiation of treatment late in the disease course [221].

In addition to IVIG, some researchers have used other strategies and therapies for polyomavirus treatment, such as drugs with broad anti-viral activity, including cidofovir, leflunomide, and ciprofloxacin, but their usefulness remains inconclusive [222,223,224,225,226,227,228,229,230]. One possible new strategy for polyomavirus treatment is to develop an efficient vaccine.

Similar to HPV vaccines, virus-like particle (VLP) vaccines have an efficient safety record and are known to induce high levels of neutralizing antibodies associated with a long period of protection against HPV infection [230]. In 2023, a study found that a multivalent polyomavirus vaccine was effective in rhesus macaques, producing neutralizing antibody titers that lasted for almost two years. The researchers also found that the vaccine boosted pre-existing immune responses and produced high-titer neutralizing antibody responses [230].

Kanse et al. conducted a study using immune informatics and molecular modeling methods to design a multi-epitope subunit vaccine targeting JCPyV in PML. They found that this vaccine enhanced immunogenicity and broadened the coverage of antigenic variants [231]. The development of a vaccine against JCPyV is complex because of the ability of the virus to persist in the human body without causing symptoms [231].

## 8. Human T-Lymphotropic Virus (HTLV)

Human T-lymphotropic virus (HTLV-I) is a retrovirus in which more than 90% of the infected individuals remain asymptomatic, facilitating its silent spread between hosts, thus persisting in humans for a long time. The infection is transmitted vertically by breastfeeding from infected mothers to neonates and horizontally through sexual intercourse and blood transfusion [232]. Unlike HIV-1, HTLV-1 is transmitted through cell-to-cell contact. In its asymptomatic form, this virus is recognized as a causative agent of essential diseases such as HTLV-I-associated myelopathy/tropical spastic paraparesis (HAM/TSP) and adult T-cell leukemia-lymphoma (ATLL) [233,234,235,236]. In addition to these diseases, a small proportion of HTLV-I carriers develop other diseases, such as HTLV-I-associated arthropathy (HAAP), cutaneous T-cell lymphoma (CTCL), Graves’ disease, uveitis, polymyositis, chronic respiratory diseases, lymphadenitis, and dermatitis [235,237].

### 8.1. How HTLV Triggers Tumorigenesis

ATLL is a CD4+CD25+ T-cell neoplasm that occurs in 3–5% of infected individuals. Among the main regulatory proteins encoded by HTLV-1 and are essential for viral persistence and latency, we have Tax and HTLV-1 basic leucine zipper factor (HBZ) [238]. These proteins are strongly involved in the oncogenic process of HTLV-1, leading to the development of serious cancers of the lymphoid lineage [239].

Oncogenesis refers to the process by which cells become cancerous due to HTLV infection. The mechanisms involved include the integration of the viral genome into host cells, activation of oncogenes, and dysregulation of several cellular processes. Understanding these mechanisms is crucial for developing early diagnostic strategies and therapeutic interventions [240].

The HTLV-1 genome comprises a positive-sense RNA of approximately 9 kb, with two copies printed on each viral particle. Upon entry into the host cell, the ssRNA genome is reverse transcribed into dsDNA and integrated into the host genome to form the integrated retrovirus provirus [241]. The viral genome contains long terminal repeats (LTRs) at the 5′ and 3′ ends. These direct repeats consist of three regions: the 3′ unique (U3), repeat (R), and 5′ unique (U5) regions. LTRs contain important elements required for viral transcription, polyadenylation, and integration. Like all retroviruses, HTLV-1 contains the structural and enzymatic proteins (Gag, Pro, Pol, and Env) in addition to five viral accessory proteins (Tax, Rex, p12, p13, and p30), which are encoded by sense transcripts derived from the 5′ LTR promoter. HBZ is the only viral accessory/regulatory protein encoded by an antisense transcript derived from a 3′ LTR promoter [242,243].

HTLV-1 infects CD4+ and hematopoietic progenitor cells. Infected cells activate the NF-κB and Akt survival pathways to prevent apoptosis and senescence. Inactivation of p53 by Tax helps in the activation of enzymatic proteins, such as cyclin-dependent kinases (CDKs), which accelerate cellular regulation and play a central role in gene regulation [244,245]. They control cell cycle progression (G1, S, G2, and M) by associating with regulatory proteins. This association is essential for the phosphorylation of target proteins that promote specific cell cycle transitions. Tax proteins contribute to genomic instability during the progression of ATLL by interfering with essential cellular processes. Tax attenuates Mad1 function, which is responsible for mitotic spindle assembly checkpoint and requires correct chromosome segregation. Tax reduces the efficiency of DNA damage repair and activates ROS production, resulting in aneuploidy (an abnormal number of chromosomes) and clastogenic damage (chromosomal breaks) [246]. In the final stages of ATLL development, when Tax expression is suppressed, other factors play a central role in malignant transformation, such as HBZ, changes in miRNAs, and inactivation of TP53INP1. These combined processes culminate in completely modified phenotypic characteristics of advanced ATLL [247].

The location of Tax in the host cell determines its function; in the nucleus, this protein participates in transcriptional activation; in the cytoplasm, it participates in the manipulation of the host’s cellular pathways; and, extracellularly, it contributes to the neuropathogenesis of HTLV-1 [248].

The Tax protein, encoded by HTLV-1, activates viral gene expression by recruiting the cAMP response element-binding protein (CREB/ATF) and CBP/p300 coactivators to the HTLV-1 LTR promoter. Furthermore, Tax endows HTLV-1 with the capacity for malignant transformation, immortalization of human CD4+ lymphocytes, and transformation of fibroblasts. Tax promotes commercialization and malignant transformation by modulating several signaling pathways, such as NF-κB, PI3K-AKT, and serum response factor; in addition to inhibiting the tumor suppressors Rb, p53, and DLG1, these are normal genes that delay cell division, repair DNA errors, or indicate when cell apoptosis should occur [247,249]. Once these genes are inhibited, cells begin to develop uncontrollably, leading to the development of ATLL [250].

The Tax1 protein is a key factor in viral oncogenesis because of its multiple biological effects that promote cellular control, deregulate the cell cycle, and facilitate malignant cellular transformation.

It has already been described that Tax is the main target of cytotoxic T lymphocytes (CTLs) [251,252]. Thus, ATLL cells often lose Tax expression via several mechanisms. First, the 5′ LTR of the HTLV-1 provirus is reported to be deleted in 39% of ATLL cases, resulting in the loss of Tax in this context [253]. The second mechanism involves nonsense mutations, deletions, and/or insertions in the tax gene in ATLL cells [254,255]. The third mechanism includes DNA hypermethylation and histone modification of the 5′ LTR of HTLV-1, which silences viral gene transcription [256,257]. Therefore, it is essential to highlight that Tax exclusively exerts oncogenic functions in ATLL.

Among the viral genes encoded by HTLV-1, HBZ is the only gene constitutively expressed in all ATLL cases. HBZ was initially identified as a binding partner of the protein CREB-2 (cAMP Response Element-Binding Protein 2), also known as ATF-4 (activating transcription factor 4). HBZ exerts multiple inhibitory effects on transcriptional regulation. It represses the CREB-mediated transcription of the cyclic AMP response element (CRE) of the cyclin D1 promoter, extending its inhibitory role in the transcription of CREB-regulated cellular genes. This repression depends on the HBZ–bZIP domain. This interaction highlights the role of HBZ in modulating cell signaling pathways and regulating gene expression, especially in processes associated with oncogenesis and the persistence of HTLV-1 in host cells [250,258].

Furthermore, HBZ interacts with p300/CBP coactivators, interfering with the association between Tax and p300/CBP, and inhibiting Tax-dependent viral transcription. This interaction is mediated by two motifs similar to LXXLL (where L represents leucine and X represents any amino acid), a specific peptide sequence found in several proteins [259]. In the context of HTLV-1, the LXXLL motif plays a crucial role in protein–protein interactions, modulating signaling and transcription pathways implicated in viral persistence and oncogenesis, present in the NH2-terminal region of HBZ, which specifically binds to KIX of p300/CBP [260]. The LXXLL motifs located in the AD domain of HBZ also allow binding to the surface of mixed-lineage leukemia (MLL) in the KIX domain. This interaction blocks the binding of MLL to the KIX domain and strengthens the binding of transcription factor c-Myb to the opposite region of KIX. These complex interactions highlight the role of HBZ in modulating transcriptional processes and HTLV-1-associated oncogenesis [252,261].

### 8.2. HTLV and Oral Cavity Cancer

Breast milk is the main route of vertical HTLV-1 transmission. Therefore, the oral cavity acts as a gateway for the virus. In vivo studies have demonstrated that mice exhibit weaker HTLV-I-specific cellular immune responses with increased viral load following oral contamination. When HTLV-infected BrMMø (breast-milk-specific antibodies) are ingested and reach the newborn’s intestinal tract, they can transmit HTLV-I virions to intestinal lymphocytes and monocytes [232,262,263].

Although they are less common, a few molecular and serological studies have detected the presence of provirus DNA and HTLV-I antibodies in the oral fluid and saliva of individuals infected with HTLV-I. However, serological and molecular diagnosis using blood samples continues to be more effective and recommended due to the type of viral replication [264,265,266,267].

Despite the difficulties in the oral detection of HTLV, some oral complications have already been associated, such as xerostomia, as the most frequent, with consequences such as hyposalivation, dysphagia, dysgeusia, opportunistic infections, cavities, and periodontal disease. In addition to these complications, Sjögren’s syndrome, an autoimmune disease characterized by dry eyes, mouth, and inflamed lacrimal and salivary glands, has already been detected and leads to fibrous changes in the labial salivary glands in patients with HTLV [268,269,270]. A 2019 study reported an association between HTLV-1 infection and advanced periodontitis being influenced by hematopoietic activity in the elderly. This may be justified because higher hematopoietic activity may help maintain endothelial function even if the magnitude of endothelial damage is severe [271,272].

### 8.3. Recent Advances and Future Perspectives in HTLV (Vaccines and/or Treatment)

Currently, there are no preventative or curative interventions available. For HTLV-infected individuals with limited treatment options, treatment is targeted according to the associated disease. Given the lack of virus-targeted treatment options, alternative strategies are required. Recent advances have been made in the development of RNA-based antiviral therapies for HIV-1 as it is an analogous retrovirus that shares modes of transmission with HTLV-1 [273]. The genetically conserved 5′ and 3′LTR regions of HTLV-1 are promising candidates for epigenetic silencing. Despite the novelty of siRNA therapeutics, many challenges, including efficient targeted delivery and clinical translation, remain to be addressed [274].

In the case of ATLL, the therapy that is based on curative intent in individuals with significant tumor volume is chemotherapy combined with antiviral therapies using PD-1/PD-L1 inhibitors, zidovudine, interferon-alpha (IFN-α), among others. Studies in Japan have highlighted the possibility of other combined therapies, such as vincristine, cyclophosphamide, doxorubicin, prednisone (VCAP), doxorubicin, ranimustine, prednisone (AMP), vindesine, etoposide, carboplatin, and prednisone (VCEP). However, they are generally not curative, which is a significant challenge in the treatment of ATLL [275,276]. Despite progress in understanding the disease, current therapies remain poor. Long-term survival has been reported, particularly among patients with indolent disease or activating mutations in the CC4 chemokine receptor. However, allogeneic hematopoietic stem cell transplantation is the only curative treatment option [277].

A vaccine for bovine leukemia virus (BLV) was recently developed. Although it is a disease that naturally infects cattle, it shares common retroviral structural proteins (Gag, Pol, and Env, as well as Tax) with HTLV-1. Therefore, this vaccine may provide a model against HTLV-1 that requires the stimulation of humoral and cytotoxic immune responses [274,278].

In Brazil, significant advances in public policies related to HTLV have been achieved recently, such as the inclusion of the virus infection in the national list of compulsory disease notifications, in addition to the incorporation of HTLV screening in pregnant women during prenatal care in the unified health system [279].

## 9. Herpes Simplex Virus Type 1 (HSV-1)

Herpes simplex virus 1 (HSV-1) is a member of the human herpes virus family and is categorized under the *Alphaherpesvirinae* subfamily. It was isolated from HSV-1 1968 by Nahmias and Dowdle based on its epidemiological, clinical, and immunological characteristics [280]. The HSV-1 genome is composed of linear dsDNA (152 kb) protected by an icosahedral capsid surrounded by a protein layer called a tegument and wrapped by an envelope containing viral glycoproteins [281]. It is estimated that 3.8 billion people under 50 years of age worldwide are infected with the virus (64.2%) [111].

Three genotypes of clinical isolates of HSV-1 have been described [282]. Genotypes were differentiated into A, B, and C, and the classification was based on DNA sequencing of the US4, US7, and US8 genes, which encode glycoproteins G (gG), I (gI), and E (gE), respectively, all of which are located in a single short region of the genome. Given the recently described genetic diversity of HSV-1, the search for associations between specific viral genetic markers and clinical symptoms is of interest [283].

HSV-1 spreads primarily through oral contact, causing infections in or around the mouth (oral or labial herpes) that can lead to serious complications, such as stromal keratitis (HSK), eczema herpeticum, disseminated neonatal disease, meningitis, and encephalitis (HSE) [284]. Several studies have suggested a link between HSV infection and neurodegenerative diseases [285,286]. HSV-1 also causes genital herpes, a characteristic symptom of HSV-2 infection [287].

Most people have no or only mild symptoms, and many people spread the virus without realizing it. Infections can cause painful, recurring blisters, or ulcers. Symptoms usually begin with tingling, itching, or burning in the areas where the sores appear. Fever, body aches, and swollen lymph nodes may also develop. The first infection is usually more severe than the recurrent episodes. Medications can reduce symptoms but cannot cure the infection [111,284].

After infection, viral replication occurs in epithelial cells. HSV-1 migrates via retrograde axonal transport to the dorsal root ganglia, where it establishes latency in neurons and persists over time. With immunological changes in the host organism, episodes of reactivation may occur, during which viral particles are transported to the axonal terminals in an anterograde manner [285,288]. The released particles promote the infection of new epithelial cells, which replicate and propagate in the environment to spread to a new host.

### 9.1. How HSV-1 Triggers Tumorigenesis

The presence of HSV-1 increases the risk of high-grade squamous intraepithelial lesions [289]. Its mechanism of action is based on the interruption of cellular DNA damage repair and the increased presence of existing oncogenes [290], such as HSV-1, HPV, and EBV, along with other premalignant and cancerous conditions that can lead to oral cancer [291], such as interference in signaling pathways, where the presence of the virus deregulates a chain of proteins that maintain normal cell function, for example, the Pl3k, Akt, and STAT3 pathways that are involved in cell survival, growth, and proliferation. HSV-1 activating these pathways causes cell proliferation and growth to be intensified, increasing the risk of the presence of mutagenic cells. Sequestration of proteins such as RPA, a protein that signals genotoxic stress via ATR, that is, it prevents signaling from occurring normally, allows the virus to replicate uncontrollably [290]. In addition to cell proliferation, there is evidence of its participation in the suppression of the apoptosis mechanism, where it prevents damaged cells from being destined for programmed death, allowing infected cells to survive and reinforcing the potential for the development of cancer cells [292].

HSV-1 can also escape the host immune system by manipulating immune cell responses, such as by interfering with the participation of the complement system and the interferon (IFN) pathway. In the complement system, glycoprotein C binds to C3b and gE binds to the Fc domain of IgG, thus blocking complement activation and antibody-dependent cellular cytotoxicity. The host virion shutdown protein (HSV) expressed from the viral gene ICP0 will produce high resistance to the host interferon system [293]. By inhibiting the IFN response, HSV-1 can promote its own replication, thus stimulating the development of mutagenic cells. It has the ability to alter and modulate the function of immune cells such as T cells, macrophages, and NK cells, suppressing their response against viral infections [294].

The relationship of HSV-1 in oral carcinoma has been studied, and its prevalence in potentially and definitively malignant lesions is approximately 30% [295]. The persistence of the virus in the oral mucosa and its ability to stimulate the synthesis and repair of host DNA during reactivation suggests that it may contribute to the progression of carcinoma [296]. Environmental stimuli activate genes associated with cell growth and/or cell death [297].

The presence of HSV-1 DNA and antigens in carcinoma cells but not in the normal mucosa of the same individual is the most concrete evidence for the involvement of the virus [298,299]. There is also evidence that HSV-1 has an even greater oncogenic potential in individuals who abuse tobacco and alcohol [300].

In vitro studies have elucidated the specific mechanisms by which HSV-1 promotes the transformation of human cells. The virus induces DNA synthesis, inhibits apoptosis, and has a mutagenic profile, all of which contribute to its carcinogenesis [301,302]. This also suggests that HSV-1 may act as a cofactor in oral oncogenesis or opportunistically infect tumors [303]. In an in vivo study, Larsson et al. showed that HSV-1 plays a carcinogenic role in the presence of tobacco and other carcinogenic chemicals [304].

Genital HSV-1 infections are known risk factors for the development of cervical cancer, and a multicenter study identified it as a cofactor in HPV-positive cervical cancer, wherein acting together with HPV may increase the risk of cancer invasion [289]. HSV-1 has also been shown to increase the risk of high-grade anal squamous intraepithelial lesions. The oral and vaginal epithelia are structurally similar [305], suggesting that HSV-1 is involved in the development of OSCC.

### 9.2. HSV-1 and Oral Cavity Cancer

HSV-1 infects the orofacial region in a generalized manner [306]. Its infectious cycle begins with a primary orolabial infection occurring mainly in the non-keratinized mucosa, such as the labial and buccal mucosa. It is less likely to develop on keratinized surfaces such as the gums, hard palate, and dorsum of the tongue [307]. After the primary infection, the virus ascends through the axons in the sensory nerves and establishes its latency site in the trigeminal ganglia.

During latency, HSV-1 will be reactivated by several factors that will then establish recurrent lesions at the site of the primary infection. It remains more exposed to the sensory neurons that innervate the outer layers of the skin and mucosa, and, therefore, are exclusively seen on keratinized surfaces of the skin and mucous membrane [307].

In contrast to primary lesions that develop in both keratinized and nonkeratinized tissues, recurrent lesions are highly restricted to keratinized epithelial tissues. This site specificity of recurrent lesions can be interestingly explained by the pathogenesis of HSV and also in the development of cancer where the injury is always occurring in the same location; by constantly stimulating the same region, the tendency for the formation of mutagenic cells increases, and the presence of keratin also plays a protective role for the virus, shielding it from immune responses [307].

The oral region has the second-largest microbiome in terms of diversity, after the intestine. It has more than 700 species of bacteria and more than 100 species of fungi and protozoa; the oral cavity can be divided into different microenvironments, each with a different microbiota composition [308]. Dysregulation in the composition of the oral microbiota is often associated with environmental factors such as smoking, high sucrose intake, and the use of antimicrobials [309].

In fact, several species are associated with periodontal diseases and also associated with an increased risk of developing gastrointestinal cancer. The mechanisms include the generation of pro-inflammatory conditions and the immunosuppression and suppression of apoptosis. In addition to driving cancer development and progression, the microbiota has been implicated in mediating resistance to anticancer therapies [310]. As more information becomes available about the relationships between pathogens present in the oral microbiota and various types of cancer, it may be possible to use its members as biomarkers for disease. The presence or absence of pathogens could guide clinical decisions regarding treatment [311].

Recurrent lesions caused by HSV-1 can lead to cancer due to constant and intense tissue damage. Oral and pharyngeal cancers are heterogeneous and affect the lips, oral cavity, and pharynx. In 1979, Shillitoe and Silverman demonstrated that individuals with oral cancer have an increased immune response to HSV-1 and concluded that the virus may be carcinogenic, suggesting that HSV-1 is a risk marker for the development of oral cancer [312]. Higher viral loads were also found in individuals with untreated oral cancer and in a control group of smokers than in healthy nonsmokers.

Starr et al., in 2001, presented a population-based study with serological evidence of HSV-1 in individuals with oral carcinoma and suggested that the presence of the virus may increase the development of SCC in individuals with adverse oral habits or HPV infection [296]. SCC is a head and neck cancer that accounts for 2% of all cancer cases worldwide [313]. There are also other studies that reinforce the association of HSV-1 with SCC [291,303].

### 9.3. Recent Advances and Future Perspectives in HSV-1 (Vaccines and/or Treatment)

The role of immunosuppressant treatment in the relationship between HSV-1 and oral carcinoma has not yet been evaluated. Future studies should evaluate whether these treatments increase the risk of severe HSV-1 infection [289,314]. It has been observed that individuals with lip cancer concomitantly with HSV-1 infection have been associated with higher mortality. Owing to the limitations of retrospective analyses of the database, there is a need for more in-depth studies and clinical trials focusing on HSV-1 seropositivity rather than clinical diagnoses as the selection criteria [289].

In individuals with HSV-1-positive tumors, a more aggressive pattern was observed, including multiple tumors with a high recurrence rate, presenting resistance to treatment, and requiring hospitalization [289]. These results encourage future studies to investigate the presence of HSV-1 in relation to tumor aggressiveness in individuals with orofacial cancer.

Current antivirals used to treat HSV infections include acyclovir, valacyclovir, and famciclovir. The metabolites of these nucleoside derivatives interfere with viral DNA synthesis by inhibiting viral DNA polymerase [315]. Among all human herpes viruses, acyclovir has the greatest in vitro activity against HSV-1 and HSV-2. Valacyclovir, a prodrug of acyclovir, and famciclovir, a prodrug of penciclovir, have greater oral bioavailability than acyclovir [315] and are administered less frequently. These drugs are generally well tolerated because they are selectively converted to active compounds in virus-infected cells.

In general, they act on the viral DNA polymerase to prevent nucleotide cleavage and viral replication [316]. Although these drugs are effective, they do not eradicate the virus and are susceptible to long-term viral resistance. Therefore, the development of vaccines is important for preventive and therapeutic approaches to treat HSV-1-induced diseases.

The leading vaccines are divided into three types: inactivated, attenuated, and subunit vaccines. However, to date, many challenges have been present, such as ensuring the control of viral replication, effectiveness of the immune response, and vaccine safety. Strategies, such as recombinant vaccines, viral vectors, and nucleic acids (DNA/RNA), are being developed through genetic engineering techniques, viral vectors, or nucleic acid molecules to ensure that HSV-1-associated antigens are presented correctly to elicit an effective immune response [317]. Although some examples show promising results in animal models and clinical trials, important issues remain to be resolved, such as immune escape from the virus, which is quite sophisticated; the durability of the immune response; and costs and accessibility on a global scale. Studies related to vaccines are progressing, and, as our understanding of vaccines advances, more research is being conducted.

As HSV-1 is an oncolytic virus, it is also used as a template to create vectors to treat certain types of cancers. Because it has cytopathic effects, its ability to destroy cells is favorable, and it can be targeted to the desired cell group [318]. The large genome makes it feasible for multiple transgenes to be modified and inserted into regions of intergenic and non-essential genes [319]. Talimogene laherparepvec (T-VEC), a derivative of HSV-1, was the first granulocyte–macrophage colony-stimulating factor (GM-CSF) approved by the FDA for the treatment of human melanoma [320].

HSV-1 is a highly prevalent virus that causes a wide range of infections in patients with cancer. Immunocompromised individuals are at greater risk of acquiring infections because of deficient immune responses. Early detection of HSV-1 is essential for initiating treatment, limiting associated morbidity, and reducing mortality. Effective antivirals are available for the treatment of diseases associated with HSV-1. It is important to continue studies involving HSV-1 as this is the only way to reduce its impact on individuals with and without cancer.

## 10. Conclusions

Oncoviruses contribute to oral cavity malignancies by mediating persistent infection, subverting host immune surveillance, and inducing oncogenic transformation via interference with cell cycle regulation, apoptosis, and genomic stability. Advances in molecular biology and microbiome research have revealed the complex interplay between oncoviruses and oral microbiota, showing how coinfections and dysbiosis amplify viral oncogenic potential. These findings enhance our understanding of virus-induced oral cancer and support the development of novel diagnostic and therapeutic strategies. Future efforts should focus on unraveling viral latency and reactivation mechanisms and the influence of the microbiome and leveraging advanced technologies to identify biomarkers, enabling early detection, personalized treatment, and improved prevention of oncovirus-associated oral malignancies.

## Abbreviations

The following abbreviations are used in this manuscript:

AIDS	Acquired immunodeficiency syndrome
AMP	Doxorubicin, ranimustine, prednisone
ATLL	Adult T-cell leukemia-lymphoma
BKPyV	Betapolyomavirus secuhominis polyoma virus
BL	Burkitt’s lymphoma
BLV	Bovine leukemia virus
CCC	Covalently closed circular DNA
CDKs	Cyclin-dependent kinases
CTCL	Cutaneous T-cell lymphoma
CTLs	Cytotoxic T lymphocytes
DAAs	Direct-acting antivirals
DLPCL	Diffuse large B-cell lymphoma
DNA	Deoxyribonucleic acid
EBNA	EBV nuclear antigen
EBV	Epstein–Barr virus
GC	Gastric carcinoma
GCF	Gingival crevicular fluid
GM-CSF	Granulocyte–macrophage colony-stimulating factor
HAAP	HTLV-I-associated arthropathy
HAM/TSP	Myelopathy/tropical spastic paraparesis
HAV	Hepatitis A virus
HBV	Hepatitis B virus
HBZ	HTLV-1 basic leucine zipper factor
HBcAg	Core antigens
HBsAg	Surface antigens
HBxAg	X proteins
HCC	Hepatocellular carcinoma
HCV	Hepatitis C virus
HHHV-8	Human rhadinovirus gamma-8
HIV	Human immunodeficiency virus
HL	Hodgkin’s lymphoma
HNSCC	Head and neck squamous cell carcinoma
HPV	Human papillomavirus
HPyV	Human polyomaviruses
HSV-1	Herpes simplex virus 1
HTLV	Human T-lymphotropic virus
IARC	International Agency for Research on Cancer
ICTV	International Committee of Taxonomy of Viruses
IFN-a	Interferon-alpha
IM	Infectious mononucleosis
IR	Insulin resistance
IRIS	Immune reconstitution inflammatory syndrome
JAK/STAT	Janus tyrosine kinase/signal transducer and transcription activator
JCPyV	John Cunningham polyomavirus
KICS	Inflammatory cytokine syndrome
KS	Kaposi sarcoma
KSHV	Kaposi-sarcoma-associated herpesvirus
LPS	Lipopolysaccharide
LTA	Lipoteichoic acid
LTRs	Long terminal repeats
MAPKs	Mitogen-activated protein kinases
MCC	Merkel cell carcinoma
MCD	Multicentric Castleman disease
MCPyV	Alphapolyomavirus quintihominis polyomavirus
MLL	Mixed-lineage leukemia
NF-κB	Nuclear factor-κB
NPC	Nasopharyngeal carcinoma
OLP	Oral lichen planus
ORFs	Open reading frames
OSCC	Oral squamous cell carcinomas
PEL	Primary effusion lymphoma
PML	Progressive multifocal leukoencephalopathy
PVL	Proliferative verrucous leukoplakia
RNA	Ribonucleic acid
ROS	Reactive oxygen species
SCT	Stem cell transplants
SSRNA	Single-stranded RNA
SV40	Simian vacuolating virus 40
SVR	Sustained virological response
T-VEC	Talimogene laherparepvec
TLRs	Toll-like receptors
VCAP	Vincristine, cyclophosphamide, doxorubicin, and prednisone
VCEP	Vindesine, etoposide, carboplatin, and prednisone
VLP	Virus-like particle
WHO	World Health Organization
c-ART	Combined antiretroviral treatment
cccDNA	Covalently closed circular DNA
pgRNA	Pregenomic RNA
sgRNA	Subgenomic RNA
